# The Mechanism of Quercetin in the Treatment of Lung Squamous Cell Carcinoma Based on a Protein-Protein Interaction Network

**DOI:** 10.1155/2022/9985160

**Published:** 2022-12-27

**Authors:** Yiping Lu, Wenfeng Zhang, Huayao Li, Cun Liu, Dandan Gao, Jing Zhuang, Ruijuan Liu, Jibiao Wu, Changgang Sun

**Affiliations:** ^1^Institute of Integrated Medicine, Medicine College, Qingdao University, Qingdao, Shandong 266071, China; ^2^College of Traditional Chinese Medicine, Shandong University of Traditional Chinese Medicine, Jinan, Shandong 250014, China; ^3^School of Traditional Chinese Medicine, Macau University of Science and Technology, Avenida Wai Long, Taipa, Macau, China; ^4^College of Traditional Chinese Medicine, Weifang Medical University, Weifang 261000, China; ^5^Clinical Medical Colleges, Weifang Medical University, Weifang 261000, Shandong, China; ^6^Department of Oncology, Weifang Traditional Chinese Hospital, Weifang 261000, China

## Abstract

**Background:**

Lung squamous cell carcinoma (LUSC) is characterized by poor prognosis and obvious limitations of therapeutic methods. The molecular target and mechanism of quercetin (QR), a natural anticancer product with extensive pharmacological activities, on lung squamous cell carcinoma is still unclear.

**Method:**

The effects of QR on LUSC were examined using cell proliferation, migration, and invasion tests. Key target genes were screened using The Cancer Genome Atlas (TCGA) database, Gene Ontology (GO)/Kyoto Encyclopedia of Genes and Genomes (KEGG) database, STRING website, topology, and prognosis analysis, molecular docking, and other bioinformatics methods for further analysis. Finally, the effects of QR on the expression of key targets in LUSC cells were detected using a cell cycle assay and western blotting.

**Results:**

Our study demonstrates that QR not only inhibits the proliferation of LUSC but also affects the invasion and metastasis of LUSC. After downloading and analyzing the TCGA database, 2150 differentially expressed genes were identified. PLK1, CDC20, and BUB1B were identified using enrichment analysis, topological network analysis, cluster analysis, and molecular docking screening. Subsequent experiments showed that QR could interfere with the cell cycle and downregulate the expression of the target gene PLK1 at the protein level.

**Conclusions:**

We found that QR not only inhibits the proliferation, migration, and invasion but also blocks the cell cycle progression of LUSC. QR downregulated the expression of the LUSC target gene PLK1 at the protein level.

## 1. Introduction

Non-small-cell lung carcinoma (NSCLC), one of the deadliest malignancies worldwide accounting for approximately 80% of all lung cancers [1], is considered the leading cause of cancer-related deaths worldwide [2]. The two main histological phenotypes of NSCLC are adenocarcinoma and squamous cell carcinoma. Lung squamous cell carcinoma (LUSC) is one of the most common types of lung cancer and originates from the bronchial epithelium [3]. Malignant epithelial tumors that exhibit keratinization and/or intercellular bridging squamous cell differentiation currently have a poor prognosis, with a 5-year survival rate of only 2%–13% [4]. At present, the main methods of treatment for LUSC include surgery, chemotherapy, and molecular-targeted therapy; however, there are no specific molecular-targeted drugs for advanced LUSC, so the traditional cytotoxic chemotherapy is still the main treatment for advanced LUSC [5]. When most patients with advanced LUSC have experienced chemotherapy resistance and recurrence; and those same patients have had to endure the toxic side effects of chemotherapy, which considerably reduces the quality of life [6]. In-depth research on the treatment of LUSC mainly focused on the development of a clinical model of LUSC [7], the construction of markers for diagnosis and prognosis [8, 9], and the optimization of the related diagnosis and treatment scheme [10, 11]. Natural products and their derivatives as natural sources of anticancer drugs have great potential in the treatment of many tumors; however, there are still some gaps in the research on natural products in LUSC, and new molecular-targeting drugs may be beneficial in the treatment of LUSC [12, 13].

Natural drugs have attracted considerable attention from drug discovery researchers in cancer therapy [14]. However, natural derivatives have already been used as adjuvants to enhance the activity of well-known chemotherapeutic drugs in a variety of applications. Natural compounds and their derivatives are playing an increasingly important role in the fields of medicine and pharmacology. The exploration of the traditional natural medicine is based on the deeper research of national pharmacology from a pharmacological basis. Some national drugs, such as *camptothecin*, a cytotoxic alkaloid extracted from *Camptotheca acuminata* [15], and etoposide, the product of the traditional Chinese herb *Dysosma versipellis* after modification, are commonly used in ant-tumor therapy [16]. Chemotherapeutic drugs have been widely developed and used for the clinical treatment of cancer [17]. Some bioactive compounds from traditional Chinese herbal plants have shown significant and promising anticancer effects, such as the traditional anticancer drug *Hedyotis diffusa*, which contains the important active ingredient, quercetin (QR) [18].

Quercetin is a natural flavonoid found in many traditional medicines that has a wide range of physiological and pharmacological activities, such as antioxidant properties, reduction of inflammation, and inhibition of tumor cell proliferation and angiogenesis [19–21]. In addition, QR has a unique advantage in the development of anticancer drugs, since Ekstrom et al. found high dietary QR intake that was negatively associated with the risk of noncardiac adenocarcinoma [22]. Several studies demonstrated the potential of QR in the treatment of lung cancer [23, 24] both as an inhibitor of the cell growth to promote apoptosis of tumor cells and as an inhibitor of gene mutation [23, 25, 26]. However, most of these studies focused on lung adenocarcinoma, and QR is used to treat LUSC, the mechanism to be further explored.

Network pharmacology can analyze the complex relationship between disease drug signalling pathway. On the basis of modern pharmacological research, it has a great potential for adjuvant therapy and drug research and development of malignant tumors. Complex mechanism research is difficult to perform in traditional pharmacology; therefore, in this study, we followed the methods of Zhang et al. [27]. A network pharmacology-based approach was carried out to study QR treatment of LUSC. The molecular targets of LUSC were explored using bioinformatics, and the target and pathway of LUSC were predicted systematically and validated by in vitro experiments. QR was found to inhibit the proliferation and migration of lung squamous cells. Additionally, QR was shown to block the cell cycle, and its target of action was verified. Quercetin has a good inhibitory effect on lung adenocarcinoma [28], but the research of quercetin on lung squamous cell carcinoma is still blank, and the current research is still lack of potential targets of quercetin on lung squamous cell carcinoma. Network pharmacology can establish the relationship between drug-component-target and disease. With this method, we obtained the chemical composition and target of traditional Chinese medicine and further screened its effective activity. Through the interaction between drug target and disease target, the mechanism of quercetin on lung squamous cell carcinoma can be explained from the level of molecular target. This research method effectively reveals the mechanism of action of QR in LUSC and provides a theoretical reference for clinical applications.

## 2. Materials and Methods

### 2.1. Cell Lines and Materials

The LUSC cell lines, NCL-H226, and NCL-H520 were obtained from the American Type Culture Collection (ATCC, USA). The cells were cultured in standard conditions (37°C, 5% CO_2_, 95% humidity) and RPMI-1640 medium (Gibco, USA) containing 1% penicillin/streptomycin and 10% fetal bovine serum (SERANA, Germany). Cells with logarithmic growth periods were used with l stage of the experiment.

### 2.2. Cell Proliferation Assessment

The effect of QR on cell proliferation was examined using the 3-(4, 5-dimethylthiazol-2-yl)-2, 5-diphenyltetrazolium bromide (MTT) assay (Sigma, USA). The cell densities of NCL-H520 and NCL-H226 cells were 5000 and 4000 per well, respectively. After 48 h of treatment with QR (30, 60, 120, 240, and 480 *μ*M), 15 *μ*l MTT was added to each well, after 4 h incubation, 150 *μ*l dimethyl sulfoxide was added. Then, the cell proliferation was evaluated by measuring the optical density (OD) at 570 nm. Use the following formula to calculate the inhibition rate of QR on cell proliferation:

(1) $$\begin{array}{c} \frac{\left(\text{OD value of experimental group}-\text{blank group}\right)}{\left(\text{OD value of control group}-\text{blank group}\right)}\times 100\%\text{.} \end{array}$$

The OD values were averaged and all measurements were repeated in at least three separate experiments, the results of which were plotted using GraphPad Prism8.0.2.

### 2.3. Wound-Healing Cell Migration Assay

Scratch marks were made with the tip of a pipette gun in NCL-H226 cell cultures when cells were inoculated into a 6-well plate, and 90% fusion was achieved within 24 h. The drug concentration selected is 0, 60, and 120 *μ*M QR-treated cells were respectively photographed at 0 h, 24 h, and 48 h, and ImageJ was used to measure and process migration distance data.

### 2.4. Transwell Cell Invasion Assay

Serum starvation of cell cultures was performed for 24 h to remove the influence of serum. The Matrigel was placed on ice in a sterile console and allowed to thaw. Thereafter, Matrigel was prepared with the serum-free medium diluted in a ratio of 1 : 8 and added to the upper chamber. Matrigel was allowed to gel by hydrating for 30 min in serum-free medium in an incubator at 37°C. Adjust the cell suspension concentrations to 1 × 10^5^ cells/ml; 100 *μ*l suspension at concentrations of 0, 60, and 120 *μ*M was added to the upper chamber, and 500* μ*l 10% FBS medium was added to the lower chamber. After 24 h of culture, the cells were fixed with paraformaldehyde and stained with 1% crystal violet solution for 15 min. The adherent cells were photographed and counted under a microscope. Three independent experiments were performed.

### 2.5. Data Collection

A total of 502 LUSC samples and 49 normal lung tissue samples were obtained from the TCGA database (https://portal.gdc.cancer.gov/) [29]. Since this information was retrieved from the TCGA database, a public dataset, no further ethical approval was required for our study.

### 2.6. Dataset Preprocessing and Differential Gene Expression Analysis

Prior to differential expression analysis, the gene expression value matrix was transformed using the log2 function in the R4.1.0 program, and these values were then expressed as log2 conversion values [30]. The PRCOMP function was then used for principal component analysis to check for potential outliers in the gene expression matrix. To ensure the quality of the data, only genes expressed in at least three samples were included for further analysis. After normalization with Edger, mRNA differential expression was studied with the LIMMA 3.40.2. package of *R* software. Adjusted *p* values were analyzed in TCGA to correct false-positive results. The differences of adj *p* < 0.05 and |log 2-fold change| ≥ 2.0 were statistically significant. The empirical Bayes program in the package was used to compare gene expression levels between lung cancer and normal tissues. For statistical analysis, the false discovery rate (FDR) correction was used to adjust the *p* value, and only those genes with *p* < 0.05 were expressed as differentially expressed genes. Hot map was performed using the PHEATMAP software package, and volcano mapping was performed using the Bioinformatics Analysis Visualization Tool Sanger Box.

### 2.7. KEGG and GO Analyses Identified Biological Processes That Significantly Enriched Modular Genes

The genes were further analyzed by David (https://david.ncifcrf.gov/) [31] for the KEGG pathway and gene ontology (GO) function [32]. Using the clusterProfiler4.0 software package on the *R* platform, the differentially expressed genes were analyzed by GO and KEGG pathway enrichment analyses, and *p* < 0.05 was set as statistically significant. We annotated the potential targets and obtained the signalling pathways in which they were involved. The point graph was generated by the “ggscatterstats” function in the “ggstatsplot” package, and the results were also submitted to Cytoscape3.8.2 for visualization in the CLUEGO module.

### 2.8. Identification of Key Genes and Analysis of Prognostic Value

To identify interactions between LUSC-related genes, we used the STRING database (https://cn.string-db.org/) to obtain protein-protein interaction (PPI) data import Cytoscape3.8.2 software for key targets. On the basis of the formed PPI network, CYTONCA plug-in and MCODE plug-in were used for topological analysis and modular analysis to identify LUSC-related candidate biomarkers [33].

To assess the impact of differential genes on relapse-free survival and overall survival, the lung cancer module of Kaplan–Meier mapper was used to investigate the relationship between differential gene expression and survival in the LUSC cohort. Kaplan–Meier plotter's main database source was TCGA and the statistical significance of this study was set as *p* < 0.05 [34].

### 2.9. Molecular Docking

To screen pivotal targets with prognostic values, the small molecular of QR structure file was extracted from the PubChem database (http://www.pubchem.ncbi.nlm.nih.gov). From the RCSB protein database (https://www.rcsb.org/), download the crystal structure of the complex. In addition, we use SYBYL-X 2.0 software to predict the possible combination pockets. Finally, PYMOL software is used for visualization.

### 2.10. Examination of the QR Effect on the Cell Cycle of LUSC Using Flow Cytometry

Cells were evenly spread on a six-well plate at a density of 5 × 10^5^ cells per well. After the cells were attached to the six-well plate at 80–90% confluency, cells were collected after the treatment with QR at concentrations of 0, 60, and 120 *μ*M (treatment groups) or culture medium + DMSO (control) for 24 h, and a cell cycle assay kit was used. The cells were incubated with the dye for 30 min and stimulated using 488 nm flow cytometry. The data were stored in groups, and the DNA content of the cells was analyzed using FLOWJ10.8.0.

### 2.11. Protein Expression Detected by Western Blotting

Collect the NCL-H520 and NCL-H226 cells incubated for 24 h at the target drug concentration. First, use RIPA buffer to split the cell membrane, and then freeze and centrifuge them at 12000 rpm for 15 min to extract protein. After protein quantification, it was transferred to the PVDF membrane and sealed with 5% skimmed milk powder (1.5 hours). Incubate the membrane with anti-PLK1 (1 : 1000) overnight at 4°C, and then select the secondary antibody of the same species for incubation (1 h). The film was exposed for different lengths of time using a gel imaging system, and the experimental data were recorded using ImageJ1.8.0 software for statistical analysis [35].

### 2.12. Statistical Analysis

Student's *t*-test or one-way ANOVA was performed for statistical evaluation. All experiments in this study were independently repeated at least three times. All data were presented as the mean and standard deviation (mean ± SD). The quantitative analysis was performed using GraphPad PRISM8.0 statistical software, and ImageJ1.8.0 software to process the data. The statistical significance was set at *p* value < 0.05 [27].

## 3. Results and Discussion

### 3.1. Quercetin Can Inhibit the Proliferation, Migration, and Invasion of LUSC

**Figure 1:**
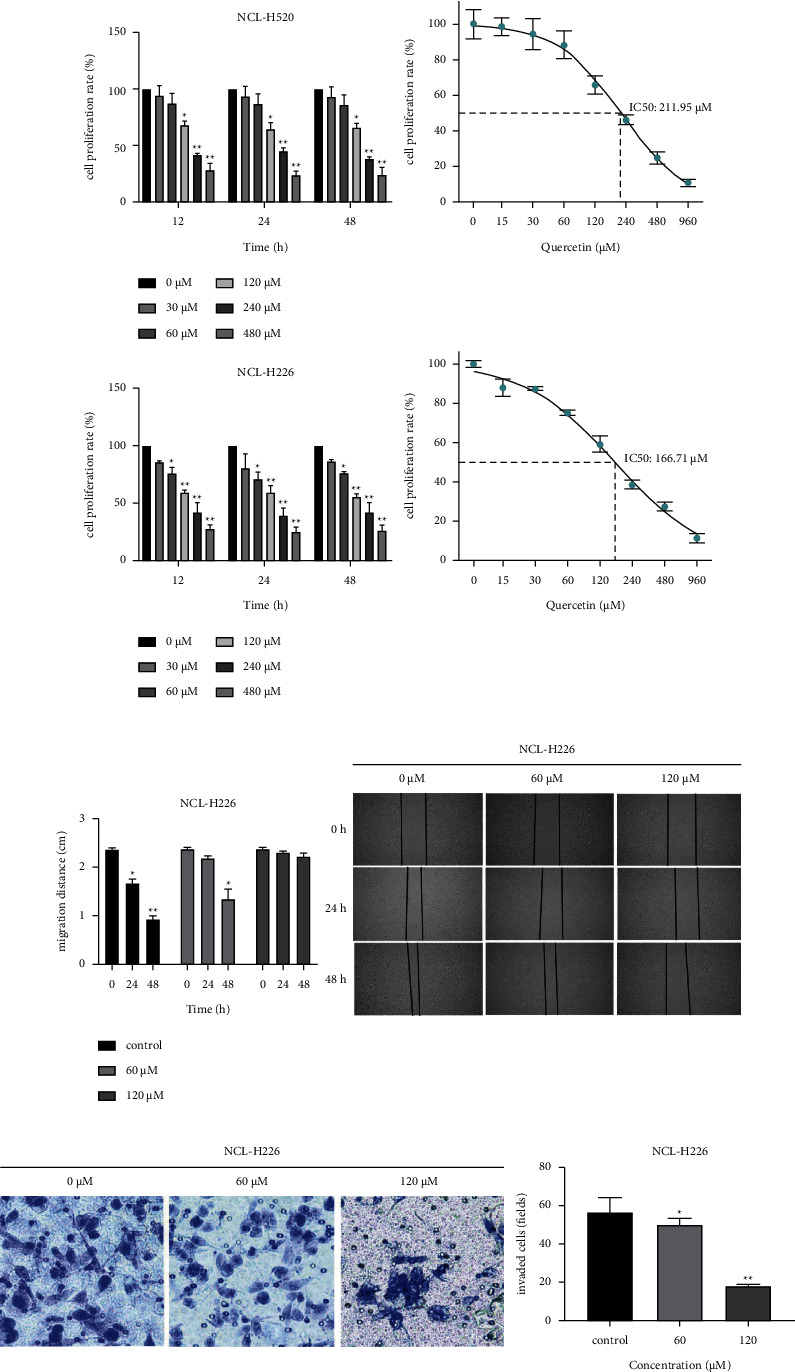
The effect of QR on the LUSC cell line activity at different time points. (a) Effects of QR on the proliferation of NCL-H520 and NCL-H226 cell lines after 12, 24, and 48 h treatment. *n* = 3 per group. (b) The antimigration effect of QR. The migration distance of LUSC cells in different groups (0, 60, and 120 *μ*M) was different after 24 h of the QR intervention with different concentrations. (c) The anti-invasive effect of QR. LUSC cells passing through Matrigel and their quantitative expressions after the intervention with QR of different concentrations (0, 60, and 120 *μ*M) for 24 h (mean ± SD; ^∗^*p* < 0.05, ^∗∗^*p* < 0.01).

The effects of QR on LUSC cell lines NCL-H520 and NCL-H226 were studied. Human LUSC cell lines NCL-H520 and NCL-H226 were treated with 30, 60, 120, 240, and 480 *μ*M quercetin for 12, 24, and 48 h. Then, MTT colorimetric assay was used to determine the proliferation. The results showed that the inhibitory effect of QR on the tumor cell proliferation gradually increased with the increase of QR exposure time. The IC50 values at 24 h for NCL-H520 and NCL-H226 cell lines were 211.95 ± 11.254 *μ*M and 166.71 ± 6.484 *μ*M, respectively (*p* < 0.01) (Figure 1(a)).

In previous studies, we found that a QR concentration greater than 200 *μ*M significantly inhibited the activity of LUSC cells. To further understand the inhibition of LUSC proliferation and its effect on the cell migration, scratch tests were performed. The scratch test is mainly used to detect the effect of drugs on cell migration and to determine the effect of drugs on the inhibition of the cell activity. Therefore, we selected 0, 60, and 120 *μ*M QR solutions for scratch tests in LUSC cell lines. The results showed that different concentrations of QR within the treatment groups versus the control group showed significant differences in cell mobility, with a statistical significance (^∗^*p* < 0.05). This demonstrated that LUSC cells have strong proliferation and migration abilities, and the scratch width in the QR-treated group was inversely proportional to the concentration of the drug, suggesting that QR has a strong inhibitory effect on the migration of LUSC cells (Figure 1(b)). Furthermore, the invasion assays show that the invasion of LUSC cells would be inhibited by QR (Figure 1(c)).

### 3.2. Identification of Differentially Expressed Genes and Differential Gene Expression Analysis

**Figure 2:**
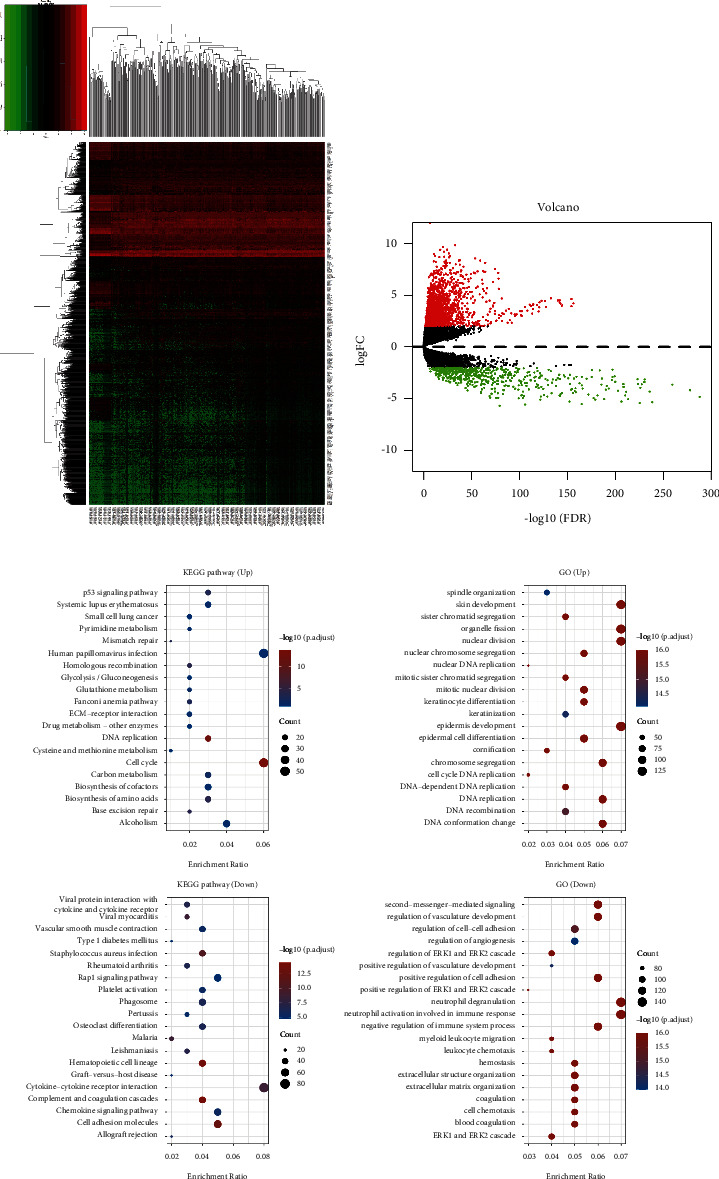
The analysis of the differential gene expression. (a) Heat map: hierarchical cluster analysis showed that 2150 DEGs could significantly distinguish the expression trend. (b) Map of volcanoes: the volcanic map of 2150 DEGs in the proteomic map between LUSC and the control group. (c) Enrichment of the function: the result of the function enrichment comes from the R package cluster profiler, in which the difference in the enrichment result is significant. The bigger the value is, the smaller the FDR value is. The number of enriched genes is expressed by the size of the dot. The larger the dot is, the more the genes are. The path of the enrichment to the significance was defined as FDR < 0.05 or *p* < 0.05.

We downloaded LUSC data samples from TCGA database. After analysis according to previous bioinformatics analysis methods, 2150 DEGs were screened, including 1572 up-regulated genes (shown in red) and 578 downregulated genes (shown in green). Hierarchical clustering analysis results showed that the 2150 DEGs could significantly distinguish the expression trends (Figure 2(a)). The volcano map was used as a graphical representation of the differential gene expression (Figure 2(b)).

### 3.3. KEGG and GO Analyses Identified Biological Processes That Significantly Enriched Modular Genes

We identify the function of target genes through feature enrichment, in which GO is a tool used to annotate genes with functions such as molecular function, biological pathway, and cell composition. The information of gene function and related high-level genomic functional molecules can be analyzed by KEGG enrichment. In order to better understand the carcinogenicity of target genes, *p* < 0.05 was set as the critical value to show that the results are statistically significant, and genes were found to be enriched in the cell cycle, human papillomavirus infection, cytokine-cytokine receptor interaction, neutrophil degranulation, and epidermal development (Figure 2(c)).

### 3.4. Identification of Key Genes

**Figure 3:**
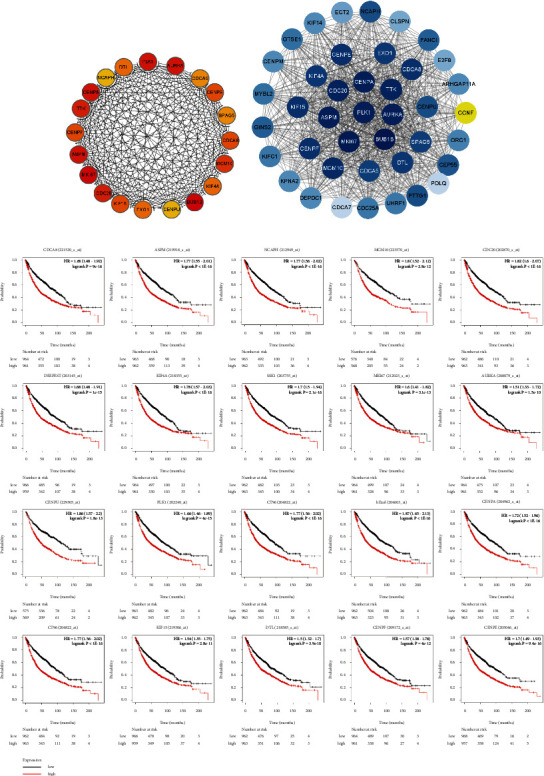
Identification and analysis of key genes. (a) The PPI network of 20 key genes: node color indicates the relationship between genes. (b) The best module selected by MCODE clustering analysis. (c) Survival analysis.

By setting the minimum confidence level of the interaction, we obtained the filtered PPI relationship network. To further screen key target genes. Next, the Centiscape plug-in was used to perform further filtering. Criteria for filtering key genes: degree >15, closeness degree >0.009, and a mean degree >60. Through the above steps, 20 target genes were finally screened, including cell division cycle 20 (CDC20), centromere protein A (CENPA), kinesin family member 4A (KIF4A), exonuclease 1 (EXO1), centromere protein F (CENPF), sperm associated antigen 5 (SPAG5), cell division cycle associated 8 (CDCA8), abnormal spindle homolog (ASPM), polo-like kinase 1 (PLK1), marker of proliferation Ki-67 (MKI67), BUB1 mitotic checkpoint serine/threonine kinase B (BUB1B), denticleless E3 ubiquitin-protein ligase homolog (DTL), aurora kinase A (AURKA), non-SMC condensin I complex, subunit H (NCAPH), centromere protein U (CENPU), minichromosome maintenance complex component 10 (MCM10), kinesin family member 15 (KIF15), centromere protein E (CENPE), cell division cycle associated 5 (CDCA5), and TTK protein kinase (TTK) (Figure 3(a)). At the same time, we used the MCODE plug-in for cluster analysis, set the node score K-core to 2, cut-off to 0.2, and max depth to 100. Based on these criteria, an important module with 41 nodes and 720 edges and a score of 36 was screened from the differential genes (Figure 3(b)). The module obtained by the above cluster analysis contains 20 candidate genes, which proved to be valuable in the subsequent analysis.

### 3.5. Critical Gene Expression and Prognostic Analysis

**Figure 4:**
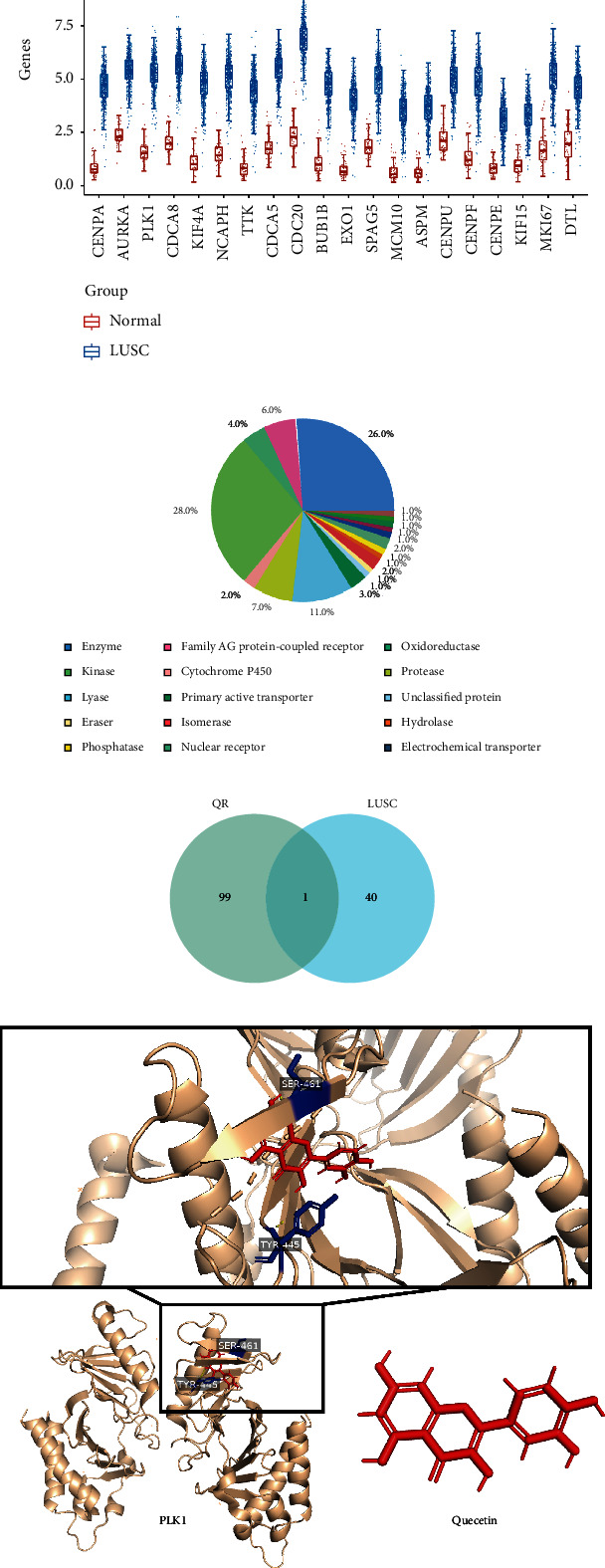
The identification of key target genes. (a) The vertical axis indicates the distribution of the gene expression, in which different groups are represented by different colors, ^∗^*p* < 0.05, ^∗∗^*p* < 0.01, and ^∗∗∗^*p* < 0.001, and the asterisk indicates the importance (^∗^^*p*^). (b) SwissTargetPrediction target of QR. (c) The Venn diagram of disease and drug targets. (d) The schematic diagram of binding sites for molecular docking of PLK1 and QR 3D models.

In order to further determine the relationship between the 20 potential target genes screened from the PPI network and the survival rate of LUSC patients, we conducted a survival analysis, and the research results showed that the 20 potential target genes are of great significance to the prognosis of LUSC patients (Figure 3(c)). Next, we will discuss the distribution of the gene expression of these target genes in tumor tissues and normal tissues, where the horizontal axis represents different genes (Figure 4(a)). Based on previous studies, the David database was used to analyze these 20 key genes. It was found that four target genes, including BUB1B, PLK1, CDC20, and TTK, were mainly enriched in the cell cycle pathway (*p* value: 7.00 *e* – 05).

### 3.6. Molecular Docking

We cross analyzed the disease target and drug target, further studied the relationship between DEGS and QR (Figure 4(b)) and crossed the previous key genes. As can be seen from the VEEN diagram, we now have a common target PLK1 (Figure 4(c)). Next, we will explore the binding site between QR and target, use SYBYL-X 2.0 to build a molecular docking model, and predict the protein affinity of the target gene through the docking score. The molecular docking score of the target gene we screened earlier is PLK1 (PDB: 5X3S, score: 6.18). PYMOL software is used for the visualization of molecular docking. QR is represented in red, protein three-dimensional (3D) structure is represented in metal color, and link sites are represented in blue (Figure 4(d)). These results show that the molecular docking of compounds is also feasible, which will help the rational design of highly effective new drugs based on the structure.

### 3.7. QR Can Block the Cell Cycle of LUSC Cells

**Figure 5:**
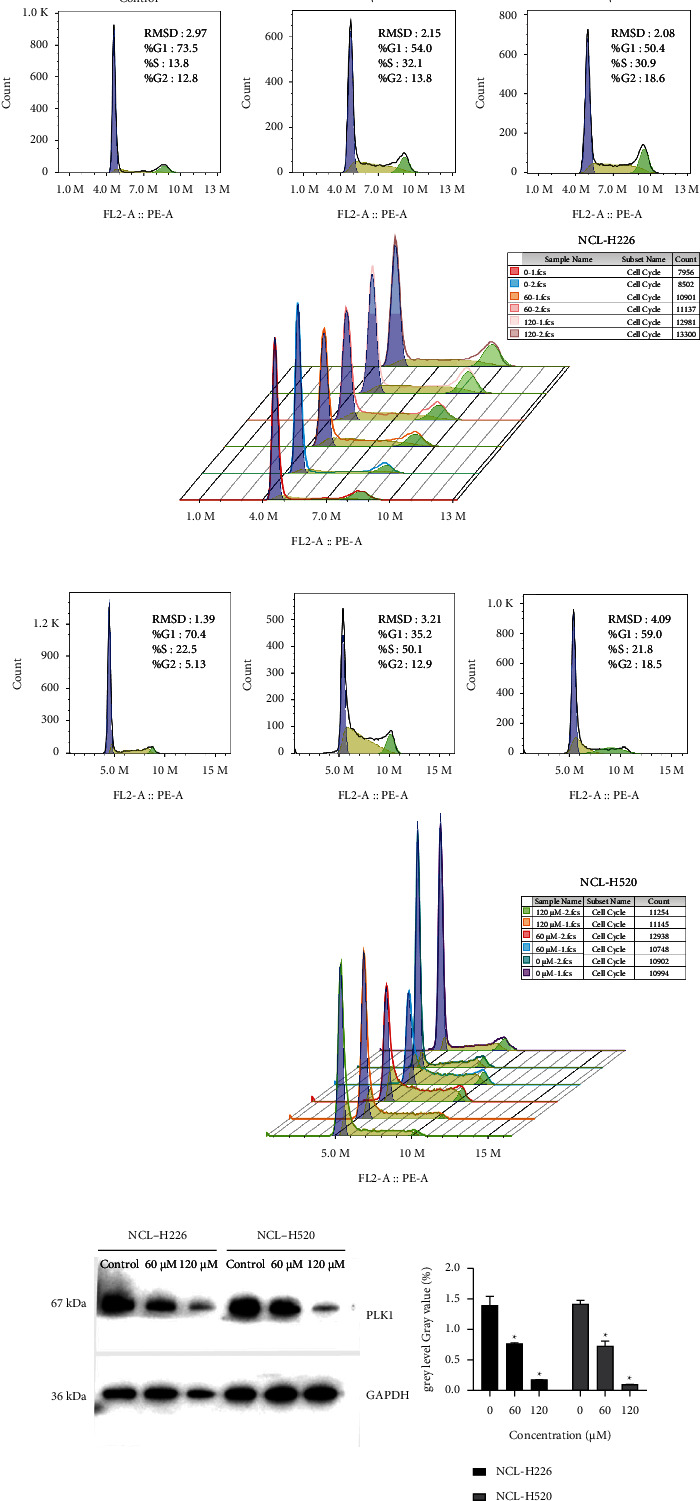
Further screening of key targets. (a) Effects of QR on cell cycle and apoptosis of NCL-H226 cells. (b) Effects of QR on cell cycle and apoptosis of NCL-H520 cells. (c) Western blot bands and quantitative analysis of PLK1 protein in two cell lines.

Based on previous studies, we know that PLK1 was enriched in the cell cycle pathway. The effects of QR on the cell cycle and apoptosis require further study. After the treatment with 60 and 120 *μ*M QR for 24 h, the cell cycle was arrested at the G_2_/M phase compared with the control group. To sum up, the flow cytometry showed that QR blocked the cell cycle progression of lung squamous cell carcinoma cell lines (Figures 5(a) and 5(b)).

### 3.8. Expression of PLK1

In order to further explore the role of QR on LUSC at the protein level, we conducted western blotting. Based on our previous research, quercetin can block the cell cycle process of LUSC cells. Some reports showed that other proteins in the cell cycle pathway will be regulated by the target gene PLK1 was screened. As we had previously predicted, we observed that QR could affect the level of identified proteins in NCL-H520 and NCL-H226 cells. The results of this experiment showed that the level of PLK1 protein decreased with the increase of QR concentration. PLK1 may be the target of QR in LUSC (Figure 5(c)).

## 4. Discussion

Squamous cell carcinoma of the lung is a malignant epithelial tumor that originates in the epithelium of the bronchial mucosal. The pathogenesis of LUSC is complex, and with the development of sequencing techniques, many studies have confirmed the role of molecular markers in LUSC [36]. In recent years, the treatment of this disease has improved, but its prognosis remains generally poor. Consequently, it is necessary to further clarify the underlying mechanisms of LUSC and to search for more effective and reliable antitumor drugs in addition to new tumor targets.

In recent years, the screening and mining of active natural products have become an important source of antitumor drug development. Natural compounds used alone or in combination have been shown to play beneficial roles in cancer treatment [37]. Traditional Chinese herbal medicines, such as ginseng and *Codonopsis pilosula*, have been proven to have anticancer [38]. For example, the active ingredients in ginseng can improve the tumor microenvironment to achieve anti-cancer effects. Because there are many kinds of components in traditional Chinese medicine, research has begun to extract and separate natural small molecular compounds from traditional Chinese medicines [39]. For example, Taxol extracted from *Taxus chinensis* can intervene in the biological process of cancer in many ways, for example, the apoptosis of breast cancer cells by activating P53, caspase-2, PLK1, and other key apoptotic proteins, the regulation of mitotic angiogenesis, and the production of ROS [40].

Quercetin, a flavonoid, is found in traditional Chinese medicines such as *Aesculus chinensis* and *Bupleurum chinensis*. QR regulates many signalling pathways, for instance the AMPK, mTOR, and PI3K-AKT pathways and can affect the growth of tumor cells and induce apoptosis [41–43]. It has *been* shown to have a variety of anticancer effects. QR can activate the *SIRT1*/AMPK signalling pathway to induce apoptosis in A549 and H1299 lung cancer cells [28], but its anticancer mechanism on LUSC remains unclear.

In this study, the effects of QR on LUSC cell lines NCL-H226 were examined by cell proliferation assessment, wound-healing cell migration assay, and Transwell cell invasion assay. In previous studies, we found that QR can inhibit the proliferation, migration, and invasion of LUSC cells. Then, we use bioinformatics and network pharmacology methods to predict key target genes. After enrichment analysis, it was found that key genes mainly participated in the cell cycle pathway. Subsequent survival analysis showed that we found that the cycle related genes CDC20, PLK1, BUB1B, and TTK were all related to the poor prognosis of LUSC patients. A cell cycle is an orderly event in which a cell grows, replicates, and divides its genome. It is regulated by many proteins and forms the basis for growth, development, and reproduction [44]. By regulating cell cycle factors, tumorigenesis and the development of gastric cancers can cause cell cycle disorders. It has been shown that STK3 promotes the tumorigenesis of gastric cancer by activating the cell cycle process mediated by Ras-MAPK [45]. Molecular docking is used to score the binding sites of QR and PLK1. PYMOL was used to map the protein quercetin binding sites to verify the results of data analysis [46]. Previous studies have shown that PLK1 is mainly concentrated in the cell cycle pathway [47]. To further verify the above conclusion, we have observed that QR has a significant effect on the cell cycle of LUSC by flow cytometry, western blot showed that QR had an effect on the protein level of PLK1 in NCL-H520 and NCL-H226 cells, and the expression of PLK1, a key target gene, was decreased compared with the control group, it suggested that PLK1 might be the target of quercetin in LUSC. QR may inhibit the development of LUSC by downregulating the cell cycle proteins associated with PLK1, thus blocking the cell cycle progression.

PLK1, a serine/threonine-protein kinase, is a cell cycle-regulated kinase and an important mitotic regulatory kinase [48]. It is mainly expressed in G2/S and M phases of the cell cycle, also can affect the entry of cells into mitosis, and the mediated phosphorylation of special products will participate in the processes of cell centrosome maturation, spindle assembly, and cytokinesis. PLK1 is also responsible for many mitotic events [49]. Overexpression of the PLK1 gene has been found in many different types of tumors and is related to the poor prognosis of cancers. Such as FBXO45 may promote IGF2BP1 activation and upregulate PLK1 in HCC [50]. In addition, PLK1 is also involved in the immune process, PLK1 can change the tumor immune microenvironment because it can promote cell proliferation and epithelial mesenchymal transformation [51] and can also increase DC maturation and rich T cell infiltration [52]. At present, PLK1 is considered an oncogene and a potential therapeutic target.

The above research shows that QR can inhibit the proliferation and migration of LUSC cell lines, and QR regulates LUSC cells by interfering with cell cycle through PLK1 gene. This study provides a molecular basis for the treatment of LUSC with QR and indirectly confirms the feasibility of our comprehensive method of using natural small molecule compounds to treat cancer. This is important because our research explores effective mechanisms by which QR may interfere with the development of LUSC. More importantly, the combination of Internet pharmacology and bioinformatics in this study was used for preliminary prediction, which was verified in vitro experimentally. The most essential element of the in vitro experimental component was the verification of the possible target screening results, thus explaining the significance of our comprehensive model. On the one hand, the in vitro experiment is an exploration of quercetin-related mechanism; on the other hand, it is also a simple verification of the integrated pharmacological model. Our next work will focus on the establishment of animal models to further explore the authenticity and validity of this model through in vivo experiments.

The results of this study may be used as a standard for effective applications of natural products and multiobjective intervention mechanisms, with the advantages of reducing clinical losses, improving efficiency and innovation.

## 5. Conclusion

Quercetin, which plays an important role in many tumors, has been used in the study of LUSC and has been shown to inhibit the proliferation, migration, invasion, and block the cell cycle. Based on the antitumor effect of QR on LUSC, we constructed a “drug-target” network. In pharmacological experiments, we demonstrated that QR downregulates the expression of the target gene PLK1 at the protein level. This model provides a new way to predict the targets of natural products and to explore the targets of LUSC genes.

## Data Availability

The microarray datasets used to support the findings of this study are available from the corresponding author upon request.
